# Investigations into the Influence of Temperature on the Tensile Shear Strength of Various Adhesives

**DOI:** 10.3390/ma16186173

**Published:** 2023-09-12

**Authors:** Arkadiusz Bernaczyk, André Wagenführ, Christian Terfloth, Jörg Lincke, Tomasz Krystofiak, Peter Niemz

**Affiliations:** 1 Jowat SE, 32758 Detmold, Germany; christian.terfloth@jowat.de (C.T.); joerg.lincke@jowat.de (J.L.); 2 Institute of Natural Materials Technology, Technische Universität Dresden, 01062 Dresden, Germany; andre.wagenfuehr@tu-dresden.de; 3 Department of Wood Science and Thermal Techniques, Poznań University of Life Sciences, 60-627 Poznan, Poland; 4 Institute for Building Materials, ETH Zurich, 971 87 Lulea, Sweden; peter.niemz@ltu.se; 5 Department of Engineering Sciences and Mathematics, Lulea University of Technology, 971 87 Lulea, Sweden

**Keywords:** wood gluing, beech, wood adhesives, temperature resistance, mechanical properties

## Abstract

The temperature resistance of glued timber, which is crucial for glued wood construction, represents a significant assessment criterion. To gain insights into this aspect, this study utilized methods such as a shear strength test in accordance with EN 302-1:2013-06 under thermal loading (from 20 °C to 200 °C), and Differential Scanning Calorimetry (DSC) to determine the glass transition temperature (Tg). An increase in thermal load resulted in a decrease in shear strength and an increase in wood breakage. A hierarchy of adhesive groups was established based on strength performance and wood failure percentage (WFP) at 200 °C. Thermoset adhesives (MF: Melamine Formaldehyde, PRF: Phenol Resorcinol Formaldehyde) led the ranking, followed by elastomer adhesives (1C-PUR: One-Component Polyurethane, EPI: Emulsion Polymer Isocyanate), with thermoplastic adhesive (PVAc: Polyvinyl Acetate) last. Thermoset adhesives further cured under heat. PUR adhesives exhibited higher strength performance at 150 °C and lower temperatures.

## 1. Introduction

The temperature resistance of glued glulam (GLT), particularly in glulam construction, is a vital evaluation criterion and largely depends on the adhesive systems used [1,2].

Bonded wooden beams, for instance, can be exposed to intense solar radiation (which causes temperatures up to about 60 °C in the roof area and in conservatories). In these situations, the bond strength should decrease only minimally and slowly, if possible. 

The topic of thermal resistance, especially in the context of timber buildings, is both highly relevant and extensively researched in the current era. As the demand for sustainable and energy-efficient construction materials increases, understanding the behavior of materials such as timber under varying temperature conditions becomes ever more critical. This knowledge helps in designing structures that are not only environmentally friendly but also durable and safe in diverse climatic conditions [3,4,5]. 

In the past, there have been studies on behavior at elevated temperatures for various wood adhesives. In the context of this topic, it is interesting to note the findings from past studies that investigated the characteristics of PVAc emulsion adhesives. These studies found that the adhesive’s bond strengths exhibited a strong relationship with their rheological properties and tensile strengths across a broad temperature spectrum, spanning from −130 to 140 °C. Interestingly, it was observed that lower temperature ranges were associated with higher shear bond strengths, while other bond strengths exhibited substantial reductions [6].

These observations were further nuanced by the suggestion that such phenomena could be the result of mechanical interlocking occurring at the wood surface, with its effectiveness varying according to the direction of stress applied to the joint. In addition, it was found that adhesives with extensive grafting exhibited enhanced bond and film strengths across the entire temperature range studied. These insights could prove invaluable when considering the performance of adhesives under diverse climatic conditions [6]. 

Furthermore, Qin (2021) evaluated the bonding strength of phenol-formaldehyde (PF) adhesive on plywood under varying hot press temperatures and durations. Qin’s study developed a mechanical mathematical model that accurately predicted the rate of bonding strength change and the maximum bonding strength of plywood. Their study found that 130 °C was the optimal temperature for plywood manufacture [7]. 

Matyašovský et al. (2019) applied DSC analysis to assess the properties of modified amine resins. The obtained results allowed for the determination of their optimal cross-linking parameters [8].

Although previous studies have explored the behavior of various wood adhesives at elevated temperatures [3,4,5], a comprehensive understanding of how different adhesive systems impact the tensile shear strength at these temperatures, particularly in the context of glulam constructions, is lacking. Moreover, existing research has rarely delved into how these effects may vary with different commonly used adhesive systems in the industry. These gaps in our knowledge pose challenges in the development of more resilient and efficient timber constructions.

To address these gaps, this study aims to comprehensively investigate the impact of elevated temperatures on the tensile shear strength of bonded wood using five distinct adhesive systems—a fiber-containing and a fiber-free 1C PUR, EPI, PVAc, PRF, and MF. These adhesive systems have been selected based on their common usage in the industry. Our objectives include understanding how these adhesive systems influence tensile shear strength under increased temperatures, and how this knowledge can inform the selection of adhesives for load-bearing timber structures. The study employs destructive testing—tensile shear strength test according to EN 302-1:2013-06 [9], due to its relevance in evaluating the performance of adhesives under practical conditions. This study continues previous research on adhesives [10].

## 2. Materials and Methods

### 2.1. Materials

#### 2.1.1. Wood

**Figure 1:**
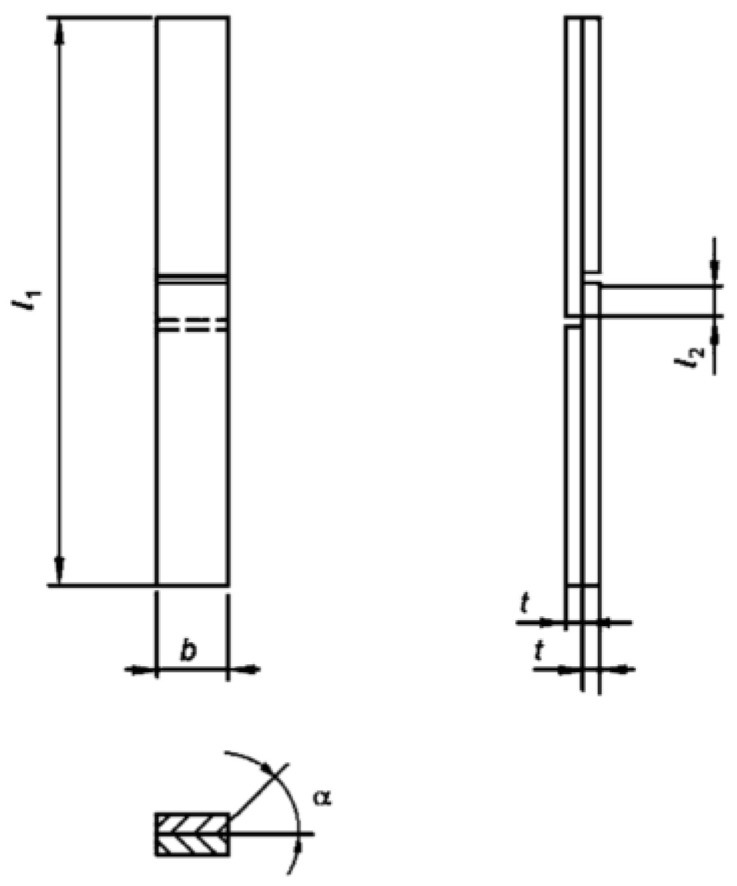
Specimen for tensile test according to EN 302-1 [9].

Beech (*Fagus sylvatica* L.) wood, with a density of 750 ± 40 kg/m^3^ at a moisture content of 12.0 ± 0.5%, was used for the bonding experiments. This choice was due to the low content of extractives (to minimize chemical interaction with the adhesives) [11]. The angle (α) between the annual rings and the glued surface ranged from 30° to 85°—Figure 1. The wood was acclimatized at 20 °C and 65% relative humidity (RH) for 30 days.

#### 2.1.2. Adhesives

Six adhesives from different manufacturers (Akzo Nobel, Amsterdam, Netherlands, Dynea AS, Lillestroy, Norway, Jowat SE, Detmold, Germany) were tested. Thin adhesive films were prepared from six distinct wood adhesives: melamine formaldehyde resin (MF), resorcinol phenol formaldehyde resin (PRF), polyvinylacetate adhesive (PVAc), emulsion-polymer-isocyanate adhesive (EPI), and one-component polyurethane adhesive (1C-PUR). 

Two PUR adhesives were examined, with the only difference between them being the addition of longitudinal polyamide fibers (approx. 5%) into PUR 1. This modified adhesive will be referred to as PUR 1F. The addition of the fibers was carried out by the adhesive manufacturer during production, using a stirring process.

The neat-adhesive films might slightly vary in behavior during the tests from bond lines due to additional reactions between wood and adhesive. It is recommended to investigate types of adhesive states to test the correlations observed [12,13,14].

Table 1 provides the main physical properties of the used adhesives, as provided in the technical data sheets from the adhesive manufacturers. The application amount of used adhesives was chosen based on the manufacturer’s technical data sheet as the most effective amount.

The properties of the tested adhesive groups that influence the choice of adhesive and its performance are shown in Table 2.

During the experimental heating process, thermosets will undergo further curing. The curing of thermosets at 20 °C typically results in vitrification, causing the crosslinking to cease. Thus, the Tg will be slightly above 20 °C, unless the reaction exhibits a substantial exotherm, such as seen in the case of MF. When the temperature exceeds Tg during the experimental heating, the reaction will resume, and the crosslinking will continue up until vitrification, unless the reaction was already complete or nearly so, as appears to be the case for EPI. 

PUR and EPI are indeed considered elastomeric materials, but they are also thermosetting polymers. Unlike thermoplastics, which can be melted and reshaped multiple times, thermosetting polymers undergo a chemical reaction when heated that sets their shape permanently. This characteristic allows them to maintain their shape and mechanical properties even when subjected to high temperatures. The soft segment of these polymers is responsible for their elastomeric properties. This part of the polymer is flexible and can deform under stress, dissipating strain energy.

PVAc, being a non-structural thermoplastic, understandably demonstrated subpar performance. Furthermore, the dual-phase morphology of PUR and EPI warrants further examination, particularly concerning their increased compliance in comparison to PRF and MF.

The parameters used during bonding are listed in Table 3 and were chosen based on the manufacturers’ recommendations in the technical data sheets to ensure optimal results.

### 2.2. Methods

#### 2.2.1. Tensile Shear Strength According to EN 302-1:2013-06

Test specimens for determining tensile shear strength were prepared following the guidelines of EN 302-1:2013-06 (Figure 1). The experiment required a minimum of 10 specimens per variant. In order to enhance the precision of the research, 20 samples per variant were conducted.

Beech boards with a thickness of 5 mm and dimensions of 130 mm × 300 mm, were utilized. The angle between the glue line and the annual ring face ranged from 30° to 85°. The solid wood samples were prepared in the same form from the same wood, but from 10 mm thick boards to avoid gluing. Beech is especially suitable for testing purposes due to its relatively homogeneous structure, high density and strength, and low extract content. Unlike spruce, commonly used in wood construction, the shear strength of beech is not too low [15].

According to this, the shear stress is particularly high in thick adhesive joints and in the edge area.

After 7 to 14 days of curing, the test specimens are tested for tensile shear strength. Since it is a relatively simple method, it is most widely used among many other test methods to investigate adhesive joints. It is often used to determine the stability of adhesives to climatic effects, to determine creep behavior (creep), heat resistance, and others [9].

The test is carried out until fracture.

The test specimens are clamped in the tensile testing machine. The force should be applied along the plane of the adhesive layer. The maximum force should be recorded.

Tensile shear strength was determined according to EN 302-1:2013-06 using a Universal Testing Machine (Zwick Z010, ZwickRoell, Ulm, Germany). The displacement for each sample was measured by the testing machine.

The rate of load increase was 0.08 mm/s and resulted in fracture within 60 ± 30 s.

Wood fracture percentage in the tested specimens was evaluated visually according to EN 302-1:2013-06. 

Test specimens were stored and air-conditioned in a normal climate until they reached mass constancy. Immediately before the test, they were conditioned at defined temperatures (20 °C, 50 °C, 70 °C, 110 °C, 150 °C, 200 °C) in a forced-air oven for 50 min, and shortly thereafter, for approximately 10 min in an oven located in the immediate vicinity of the tensile testing machine. 

##### Temperature Curves during Heating

On-line measurement of temperatures in adhesive joints was performed with a Measuring Device (ALMEMO 2590-9, Ahlborn, Germany) using thermocouples (NiCr). The accuracy of temperature determination was ±0.6 °C. The thermocouples were drilled into the adhesive joints and sealed with conductive paste.

#### 2.2.2. DSC

DSC examinations were carried out using a Differential Scanning Calorimeter (DSC 204 F1 Phoenix^®^, NETZSCH, Selb, Germany). The analyses were performed on cured adhesive films with a mass of 7.3 ± 0.1 mg using the heat-cool-heat method. The DSC analyses were conducted under the following conditions:

- Heating rate: 20 °C/min,
- Temperature range: −80 °C to 200 °C.

For the measurements, static purge gas of nitrogen (N_2_) was used to minimize interactions with the samples.

DSC investigations enable the recording of endothermic and exothermic effects. The transition from an energy-elastic to an entropy-elastic state is defined as a glass transition. This transition represents the softening of physical bonding forces in plastics. The glass transition temperature, Tg, is used to characterize the glass transition. The glass transition depends on the chemical structure, but also on the degree of curing of plastics. Tg is often used to characterize the softening of adhesives. The glass transition region is particularly pronounced in amorphous thermoplastics. It is less pronounced in semi-crystalline thermoplastics due to the crystalline fraction content [16,17].

An increase in the crystallinity of plastics is associated with increased tensile strength and a high Young’s modulus. Therefore, semi-crystalline polymer adhesives are particularly suitable for higher temperature stresses [18]. 

**Figure 2:**
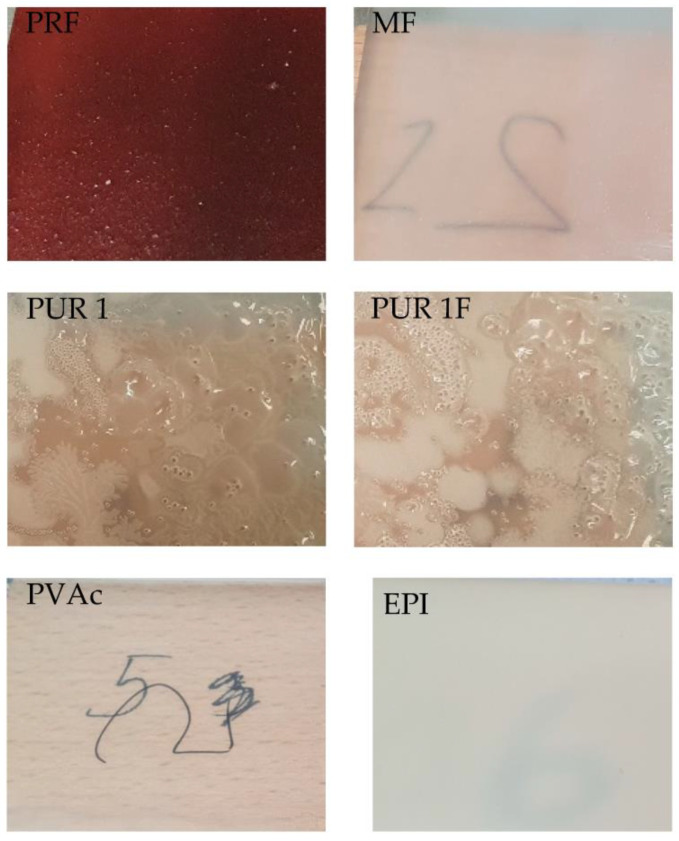
Images of adhesive films prepared on glass plates for DSC measurements.

The samples were prepared using a 200-micron applicator, films were cast over glass plates—Figure 2. 

## 3. Results

### 3.1. Shear Strength

Temperatures in adhesive joints were measured online throughout the storage in the ovens and during the testing on the tensile testing machine. Accordingly, some differences were found between the measured joint temperature during the storage in the oven and during the execution of the test—Table 4. 

These temperature changes indicate a complex interaction between the adhesive’s properties, the heating process, and the subsequent mechanical testing. The specimens were stiffening during the test. Such variations demand careful interpretation when evaluating the adhesive’s performance under different temperature conditions [19,20,21].

**Figure 3:**
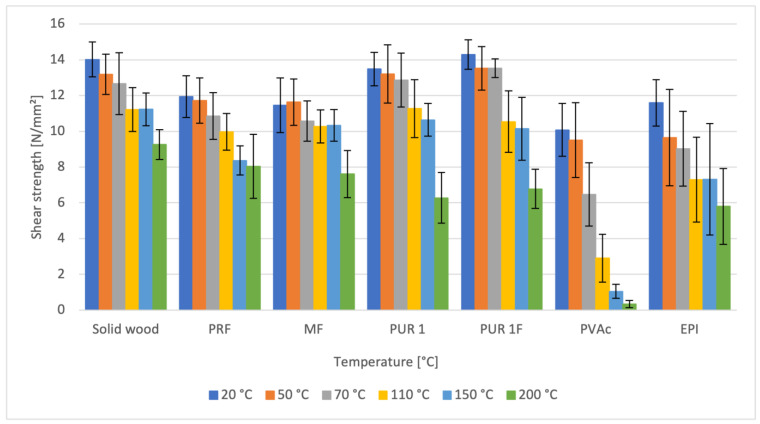
Comparison of Shear Strength for Solid Wood and Bonded Samples with Various Adhesives at Different Temperatures (The error bars indicate standard deviation).

**Figure 4:**
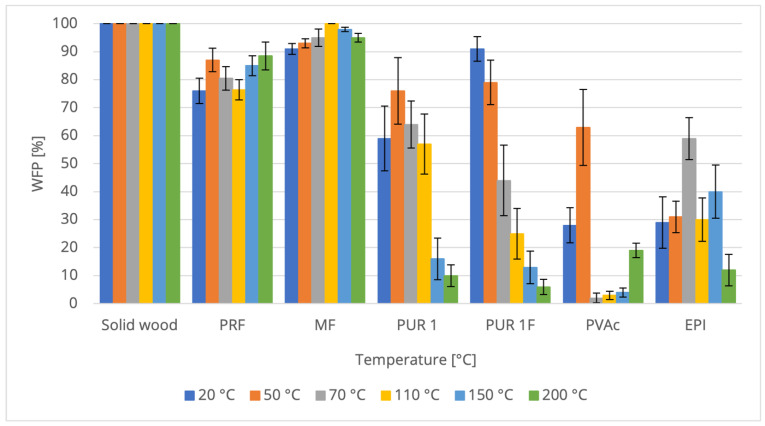
Comparison of Wood Failure Percentage for Solid Wood and Bonded Samples with Various Adhesives at Different Temperatures (The error bars indicate standard deviation).

Analysis of the results (Figure 3 and Figure 4) reveals a reduction in the tensile shear strength of wood–adhesive compounds with increasing temperature. The results exhibit a large standard deviation, making it challenging to identify clear differences. However, a decreasing trend for elevated temperatures can be observed. This impacts the decrease in wood strength as described earlier, as well as the softening of adhesive joints under heat treatment. The recorded glass transition temperatures described in Section 3.2 confirm this tendency.

Significant differences were observed between the investigated adhesive systems, as confirmed by statistical analysis using independent two-sample t-tests. In the temperature range of 20 °C to 200 °C, the reduction in the tensile shear strength of wood–adhesive joints was as follows:

- Solid wood: 34% (significant, *p* = 4.482 × 10^−19^)
- PRF: 33% (significant, *p* = 7.165 × 10^−10^)
- MF: 34% (significant, *p* = 2.478 × 10^−10^)
- PUR 1F (with fibers): 53% (significant, *p* = 6.675 × 10^−21^)
- PUR 1 (without fibers): 53% (significant, *p* = 6.027 × 10^−25^)
- PVAc: 96% (significant, *p* = 1.172 × 10^−27^)
- EPI: 50% (significant, *p* = 1.046 × 10^−12^)

Examining the achieved values of tensile shear strengths reveals that the PUR adhesives, on average, exhibit the highest temperature resistance in the temperature range from 20 °C to 150 °C amongst all the tested adhesives. At 110 °C, the difference in shear strength between PUR and other adhesives is statistically significant (*p* < 0.05). At 150 °C, the difference in shear strength between PUR and MF is not statistically significant (*p* > 0.05), indicating that MF appears equivalent to PUR in this temperature range considering the variability in the data. The PRF, MF as thermoset adhesives, and EPI as an elastomer achieve slightly lower strengths in this temperature range compared to the two 1C-PURs.

The highest values of shear strength at 200 °C are observed for the following thermoset adhesive systems: PRF: 8.05 N/mm^2^ and MF: 7.61 N/mm^2^. For the elastomeric systems, the shear strengths at 200 °C are lower: PUR with fibers: 6.28 N/mm^2^, PUR (fiber-free): 6.78 N/mm^2^, EPI: 5.80 N/mm^2^. For the thermoplastic PVAc, a particularly low shear strength of 0.38 N/mm^2^ is noted. The high strength values of the two thermoset adhesive systems (PRF and MF) suggest they may have higher temperature resistance—Table 5.

### 3.2. DSC

**Figure 5:**
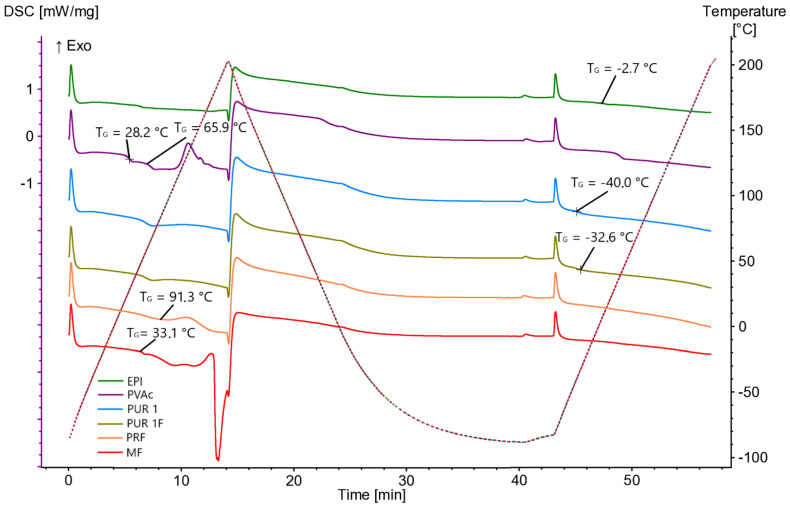
DSC Measurements for Six Investigated Adhesive Systems During First Heating, Cooling, and Second Heating.

DSC analyses are shown in Figure 5.

The samples were pressed at 20 °C, therefore, thermoset systems such as PRF and MF are expected to exhibit Tgs slightly above 20 °C. For MF, the endothermic reaction (residual cure) starts near 30–40 °C, which corresponds to the Tg of MF (33.1 °C in Figure 5). The reaction could continue during experimental heating, implying an additional cure for all heated tests. PRF exhibits a self-heating effect, with the exotherm starting near 90 °C, which corresponds to the Tg of PRF (91.3 °C in Figure 5). Additional curing of PRF would be pronounced only in the three highest temperature treatments.

Both PUR adhesives might be expected to exhibit subambient Tgs, in the range of −70 °C to −20 °C due to the soft segment. After cooling down and during the second heating of PUR adhesives the Tgs were achieved. Tg of PUR 1 is −32.6 °C and Tg of PUR 1F is −40.0 °C.

PVAc, the only nonstructural adhesive used in this study, exhibits two distinct Tgs at 28.2 °C and 65.9 °C. The higher Tg is likely from a non-PVAc component. The exotherm starting near 100 °C probably reflects thermal degradation. As PVAc is nonstructural with a very low Tg, the chemical reaction occurring during experimental heating does not significantly impact its performance.

The EPI adhesive should exhibit two Tgs, one from the latex additive, and one from the resulting hard segment. The EPI likely exhibits a low Tg of −2.7 °C, and residual cure starts at 35–40 °C. Some additional cure occurs during experimental heating, but not much.

In summary, for all adhesives except PVAc, the Tgs are expected to increase during the experimental heating if that heating exceeds the Tgs estimated in Figure 5. These are estimations as the presence of wood can change the result.

## 4. Discussion

A decrease in solid wood strength (reduction of tensile shear strength by 34%) is observed with increasing temperature within the investigated range from 20 °C to 200 °C. This aligns with Wagenführ’s data indicating that the tensile shear strength of solid wood reduces by about 8% (structural timber dimensions) and around 11% (for small samples) in the temperature range from 20 °C to 100 °C [21]. This has been confirmed by multiple studies conducted by Plath and Kollmann [23,24,25]. 

Additionally, it is important to note the role of simple thermal effects in these observations. This can be attributed to the Brownian motion or increased polymer mobility, which causes a slight decline in stiffness due to thermal effects, separate from any degradation. The influence of these thermal effects on the behavior of wood and adhesives under different temperature conditions adds another layer of complexity to the analysis.

The reasons for the strength decrease are explained below. During heating, extractives undergo significant changes. In this moderately low thermal treatment range, only minor changes occur in lignin, cellulose, and hemicellulose.

Lignin is the least thermally stable polymer in wood, followed by hemicelluloses. Wood softening is highly moisture-dependent. The traditional use of Klason lignin determination for analyzing lignin behavior during heating treatments can be misleading. Lignin monomer analysis, such as nitrobenzene oxidation or thioacidolysis, provides a more accurate understanding of lignin behavior. The viscoelastic properties of hydrothermally treated woods vary due to structural alterations of cell wall polymers and changes in their interactions. Considerable changes in amorphous polymers contribute to these variations. Hardwood lignins are more sensitive to hydrothermal treatment than softwood lignins, with the rigidity decrease primarily due to degradation of β-O-4 linkages. The most severe treatments induce extensive degradation of β-O-4 structures and selective loss of β-1 structures. There is a substantial loss of arabinose and galactose, branch sugars, during hydrothermal treatment. After heating, the yield of β-O-4 linkages in unheated oak is reduced by 29%, indicating substantial changes to the lignin structure. Lignin’s aromatic structure is often cited for extreme thermal stability, but the propyl sidechain of lignin undergoes extensive thermochemical change due to its direct attachment to the aromatic ring [26,27,28,29,30,31,32,33].

According to Hänsel (2015), the loss of wood strength occurs due to the partial degradation of certain cell wall components during heat treatment [34].

The research conducted by Schnider, Niemz, and Hurst (2008) confirmed that heat treatment of wood leads to a substantial reduction in its density, which subsequently results in a significant decrease in flexural strength and flexural modulus [35]. Numerous studies prove that, along with the reduction of strength values, the hardness of beech wood is also substantially reduced after thermal treatment [36,37].

The PVAc adhesive investigated exhibits a significant reduction in strength already above 70 °C, a result that is backed by statistical analysis. This corresponds to a marked change in the adhesive’s strength. As a pronounced thermoplastic system, it softens considerably under the influence of heat. This leads to oscillation of the polymer chains, resulting in an increase in their spacing, and ultimately, softening. In the case of thermosetting adhesives (PRF, MF), often referred to as duromers, covalently bound joints are formed during the curing process, which are 10 to 100 times stronger than physical interactions. These exhibit markedly superior thermal resistance. It is important to note that once these adhesives have fully cured, additional covalent bonding does not occur unless the adhesive is heated above its glass transition temperature, which is the case for all types tested except PVAc.

Covalent bonds, such as those found in superglue adhesives, offer the strongest interactions (150 to 950 kJ/mol) but require an extended time (minutes to hours) or assistance by external stimuli (e.g., ultraviolet light and heat) to cure and achieve appreciable adhesion. Unlike adhesions based on molecular interactions, such as van der Waals forces (2 to 15 kJ/mol) and hydrogen bonds (10 to 40 kJ/mol), covalent bonds are not reversible once cured. 

Chemically cross-linked elastomeric adhesives, such as EPI and PUR, form structures that exhibit a dual-phase morphology. This includes a soft segment with a low Tg and a hard segment with a high Tg. The unique properties of these adhesives can be attributed to this dual-phase structure.

A pronounced relationship is observed between the rise in temperature and the degradation of strength and WFP. However, it should be noted that this observation specifically pertains to the tested PUR systems and may not universally apply to all PUR variants. The addition of polyamide fibers into PUR 1 did not meaningfully alter the strength behavior of the bond at room temperature, or under thermal stress in the range up to 200 °C. The same tendency was not observed in WFP.

It is only at 200 °C that the tensile shear strength of PURs falls below 7 N/mm^2^, and starting from 150 °C, the WFP achieves lower values compared to the thermosetting adhesives (16% for PUR with polyamide fibers and 13% for fiber-free PUR).

It must be mentioned that tested adhesives were not specially developed for high temperatures. In further investigations, new heat resistant PUR adhesive will be tested. Measured temperatures correspond with the literature values [38,39].

The determination of glass transition temperature is not precise, as each person may interpret the graph slightly differently.

## 5. Conclusions

The temperature resistance of bonded glulam used in glued laminated timber construction is one of the most critical assessment criteria and largely depends on the adhesive system used. Particularly in the case of intensive solar radiation, the outer glued joint of glulam is exposed to particularly high temperatures, leading to high shear stress. Therefore, understanding the influence of elevated temperatures on the tensile shear strength of bonded wood with different adhesive systems is of utmost importance.

Shear strength of native and bonded wood and WFP are dependent on all investigated adhesive systems (PRF, MF, 1C-PUR, EPI, and PVAc), thermal stress level, and type of adhesive.

The highest strengths and WFP of bonded wood in the highest temperature range of 200 °C are obtained using thermosetting resins (PRF and MF). The group of elastomeric products (1C-PUR and EPI) achieves slightly lower strength (by 61%) and significantly lower WFP (by 55%).

The thermoplastic system (PVAc) shows by far the lowest strength values (0.34 N/mm^2^) and WFP (20%) at high temperatures. No significant differences are observed between the strength values under thermal stress for the fiber-containing (PA) and fiber-free 1C-PUR.

The glass transition temperature values for the thermoset system (PRF and MF) are distinctively the highest. This correlation extends to the highest strengths and WFP achieved in the shear strength test under thermal load. Tg for elastomeric products such as 1C-PURs and EPI), as well as thermoplastic PVAc are significantly lower.

However, it should be noted that these findings are specific to the tested 1C-PUR. It is worth mentioning that newer adhesives have emerged on the market with higher resistance to elevated temperatures and fire-retardant properties. These adhesives have been proven to yield better results under such conditions, and it is recommended to include them in future testing to further explore their performance under thermal stress.

## Figures and Tables

**Table 1:** Main physical properties of the used adhesives provided in the technical data sheets, according to the adhesive manufacturers.

Adhesive	Density [g/cm^3^]	Viscosity [mPa·s]	Solid Content [%]	Open Time [min]
PRF	1.15 ± 0.02	950 ± 550	58 ± 3	180
MF	1.21 ± 0.05	12,500 ± 5300	64 ± 3	10
PUR 1	1.15 ± 0.05	15,500 ± 2500	100	25
PUR 1F	1.15 ± 0.05	13,500 ± 2500	100	25
PVAc	1.05 ± 0.05	5000 ± 2000	49 ± 2	10
EPI	1.5 ± 0.05	11,000 ± 2000	60 ± 2	10

**Table 2:** Main properties of investigated adhesives groups [7].

Adhesive	Structural	2-Part Mix	Water-Based	Thermoset	Structure	Compliant?	Solids Content	Cure during Exp. Heating?
PRF	yes	yes	yes	yes	homo	no	58	yes
MF	yes	yes	yes	yes	homo	no	64	yes
PUR	yes	sometimes	no	no	dual-phase	yes	100	yes
PVAc	no	sometimes	yes	no	latex film	yes	50	no
EPI	yes	yes	yes	yes	dual-phase	yes	60	very little

**Table 3:** List of adhesives and parameters used.

Adhesive	Application Amount [g/m^2^]	Pressing Time [min]	Pressing Temperature [°C]
PRF	180, both sides	300	20
MF	200, both sides	360	20
PUR 1	200, one-sided	80	20
PUR 1F	200, one-sided	150	20
PVAc	185, one-sided	45	20
EPI	160, one-sided	45	20

**Table 4:** Temperatures of adhesive joints before and during shear strength testing.

Set Temperature in the Oven [°C]	Joint Temperature in the Oven [°C]	Joint Temperature during Shear Strength Test [°C]	
		Beginning	End
50	48	49	42
70	68	67	53
110	107	108	82
150	149	145	122
200	200	198	147

**Table 5:** Temperature resistance evaluation adapted on ASTM D7247 [22].

Variant	Average Shear Strength at 20 °C [N/mm^2^]	Lower 95% Confidence Interval at 20 °C [N/mm^2^]	Average Shear Strength at 200 °C [N/mm^2^]	Lower 95% Confidence Interval at 200 °C [N/mm^2^]	$\frac{\tau \;at\;200\;{}^{\circ}C}{\tau \;at\;20\;{}^{\circ}C}$
Solid wood	14.02	13.60	9.26	8.90	0.62
PRF	11.93	X	8.05	x	0.67
MF	11.46	X	7.61	x	0.66
PUR 1	13.48	X	6.28	x	0.47
PUR 1F	14.29	X	6.78	x	0.47
PVAc	10.08	X	0.38	x	0.04
EPI	11.59	X	5.80	x	0.50

## Data Availability

Not applicable.
